# Guidewire knot formation with peripherally inserted central catheter

**DOI:** 10.1002/ccr3.3662

**Published:** 2020-12-15

**Authors:** Hiroaki Saito, Tsuyoshi Suda, Yoji Nishida

**Affiliations:** ^1^ Department of Internal Medicine Kanazawa Municipal Hospital Kanazawa Japan; ^2^ Department of Surgery Kanazawa Municipal Hospital Kanazawa Japan

**Keywords:** cardiovascular disorders, Vascular surgery

## Abstract

The formation of guidewire knots during PICC insertion is an extreme rare complication. Forced insertion or withdrawal of a knotted guidewire may cause tearing of the veins. Surgical guidewire removal is required due to risk of vascular injury.

## Graphical abstract

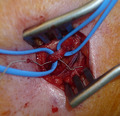

## 1 CASE HISTORY/EXAMINATION

There are various complications of PICC, bloodstream infection is well known, but the formation of guidewire knots during PICC insertion is rarely reported. Considering the risk of vascular injury in our case, surgical guidewire removal was performed. Although rare, it is necessary to recognize proper treatment for these complications.

**Figure 1:**
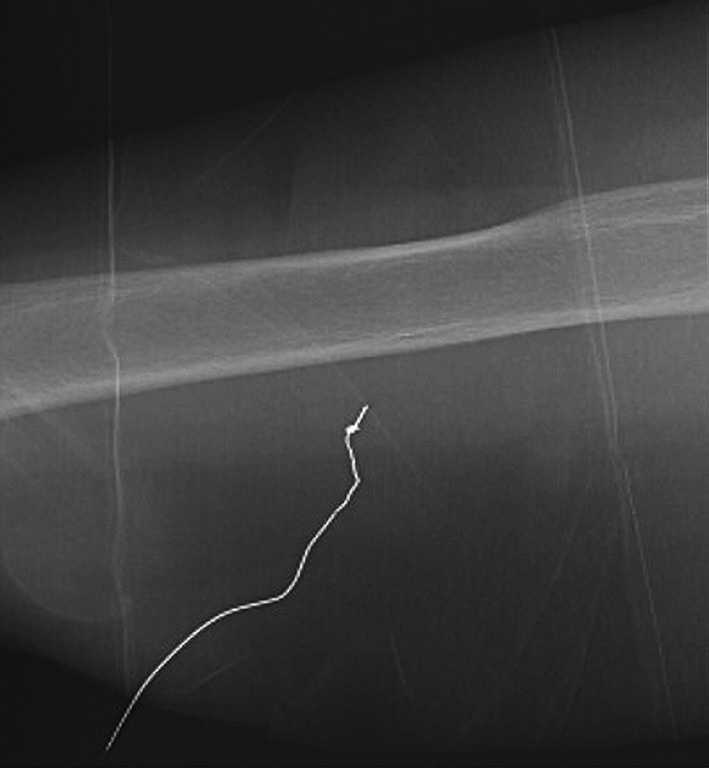
Radiograph indicating a knot‐like appearance at the tip of the guidewire

An 85‐year‐old woman was admitted to our hospital for treatment of a liver abscess. To ensure nutritional intake following the patient's loss of appetite, a central catheter was needed for parenteral nutrition and a PICC was determined to be the best choice for this patient. We used the 4‐Fr 1‐lumen Arrow PICC (Arrow International; Reading, PA, USA). The guidewire had a diameter of 0.46mm and a length of 80cm. The right basilic vein of the forearm was selected and punctured using an ultrasonic guide, and a guidewire was inserted into the vein with the modified Seldinger technique. At approximately 10 cm, the guidewire encountered strong resistance, and we were unable to remove the guidewire from the patient's body. Radiography indicated that a knot had formed at the tip of the guidewire (Figure 1).

**Figure 2:**
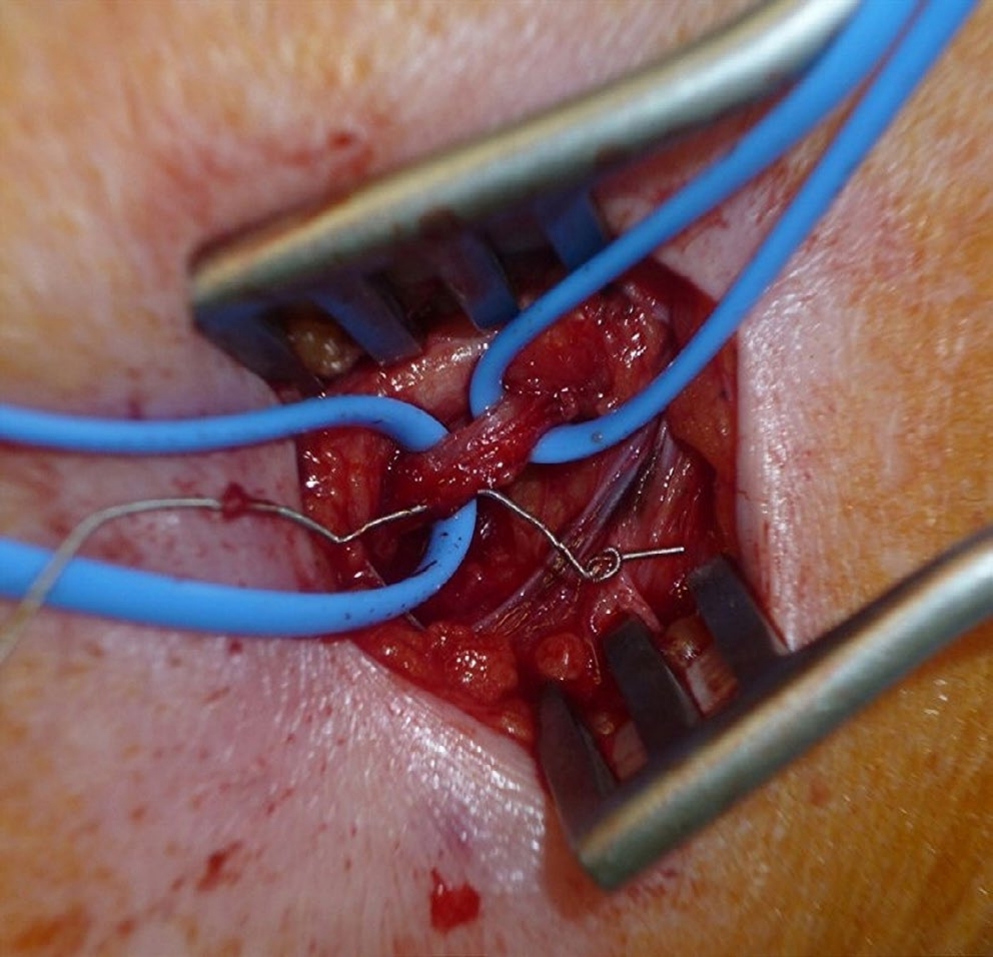
Intraoperative findings showing the guidewire penetrating the vein wall and forming a knot outside the vein

As there was risk of crushing or breaking from forced removal of the guidewire, surgical intervention was performed under local anesthesia for safe removal. Intraoperative findings revealed that the guidewire had penetrated the vein wall and formed a knot outside the wall (Figure 2). No complications occurred after surgery. Although central venous access catheters are known to cause various complications, this is, to our knowledge, the first report describing knot formation of the guidewire during PICC insertion. Previous reports have shown that forced insertion or withdrawal of a knotted guidewire may cause tearing of the veins and that surgical intervention is appropriate in such cases.^1^, ^2^ Although this is a rare complication, it is important for clinicians to know that the guidewire can become knotted and be aware of appropriate treatment methods such as surgical intervention.

## CONFLICT OF INTEREST

There are no conflicts of interest to declare.

## AUTHOR CONTRIBUTIONS

HS contributed to the editing of the manuscript and preparation of the figure. TS cared for the patient, conducted the literature search, edited the manuscript, and prepared the figure. YN cared for the patient, contributed to the editing of the manuscript and provided expert opinion on surgery.

## INFORMED CONSENT

Written consent to publish was obtained from the patient and her family.

## Data Availability

The data that support the findings of this study are available on request from the corresponding author. The data are not publicly available due to privacy or ethical restrictions.
